# The Royal Portsmouth Hospital

**Published:** 1914-08-01

**Authors:** H. F. Lapthorn

**Affiliations:** Chairman of the Committee of Management. Portsmouth


### The Royal Portsmouth Hospital.

*To the Editor of* The Hospital.

Sir,—In your last issue you pillory the Portsmouth Hospital in a series of general charges which, with your permission, I will epitomise :

1. We are a hospital where some of the worst features of the old system of patronage prevail.
2. The hospital is not generally popular.
3. It lacks, from the administrative and managerial point of view, initiative and knowledge.
4. It suffers from the evils attending cliqueism in the management.
5. It is, administratively, notoriously behind the times, and, not unnaturally, languishing for want of adequate support.

And you condemn strongly :

6. The condition of our pathological department, including our mortuary, and
7. The admission committee.

I will deal first with number six. We haven't a pathological department, so how its condition can be such as you hint at I am at a loss to understand. Some months back we decided to establish one, and voted the money for it, but, so far, have not succeeded in obtaining the services of a medical man to take charge of it. Meantime, we are continuing to send our pathological work to London.

Now, as regards our mortuary. You can tell us nothing about its condition which we don't already know and realise. We have, since 1897, raised and spent in modernising our buildings nearly £50,000 (of which one-half has been spent in the last nine years). Not having enough money to do the whole, our first care was those parts which received the living. Further, we should by now have had a new mortuary through an anonymous friend but for a practical difficulty which has blocked us for a time.

I refrain from saying anything for the present about your travesty of the function and the *modus operandi* of our admission committee.

I am certain our subscribers and the public here have heard of your charges with considerable surprise, and are not prepared to accept them as true on your *ipse dixit*, or that of your anonymous informant. You may think it fair to circulate anonymous charges without making any attempt to find out for yourself whether they are true. All those whose good opinion we value will, I am convinced, agree with me that it is grossly unfair.

You say you have called attention to the condition of the Portsmouth Hospital more than once. The only occasion I remember was about three years ago. You then made some of the same charges which you now repeat—among them, want of confidence on the part of the subscribers resulting in insufficient financial support, and bad management. I replied to you, and, though I cannot put my hand now on the correspondence, I have before me a copy of our local newspaper—the *Portsmouth Evening News*—which referred to it in these terms :—" In an Editorial note The Hospital accepts Mr. Lapthorn's corrections, and all it now urges is that the ordinary income is much too small. Upon this point we agree with our contemporary, but the insufficiency is not due either to lack of good will, confidence, or energy."

And now I challenge your anonymous informant—obviously a Portsmouth man, and apparently a medical man—to come out into the open, and he shall then have his answer. If he does this, he will have done something—though rather late—to remove the reproach that he is " willing to wound, but yet afraid to strike."

If, however, he still prefers to hide behind you, then I challenge you to prove his charges for which you have assumed responsibility.—Yours truly,

H. F. Lapthorn, Chairman of the
Committee of Management.

Portsmouth, July 27, 1914.

[This letter of Mr. Lapthorn does not meet the points we urged. We took infinite pains to ascertain the actual position and atmosphere of the Royal Portsmouth Hospital as it in fact is, and had solid grounds for the criticisms contained in the article. We were, however, careful to state " we had no doubt whatever that the members of the committee are conscientious according to their lights." Mr. Lapthorn's letter shows it is " their lights " which are faulty and need scrapping. We hope this may be recognised, for then they should be enabled to see what is wrong, and not continue to cling with dangerous tenacity to ancient methods and a defective system. It may be noted, too, that whilst Mr. Lapthorn admits the grave defects of the mortuary and its adjuncts, he fails to realise that these are necessary factors in every pathological unit, although he emphasises their value by stating that the pathological work is at present being sent to London.—Ed. The Hospital.]

